# Molecular prediction of adjuvant cisplatin efficacy in Non-Small Cell Lung Cancer (NSCLC)—validation in two independent cohorts

**DOI:** 10.1371/journal.pone.0194609

**Published:** 2018-03-22

**Authors:** Ida Kappel Buhl, Eric Santoni-Rugiu, Jesper Ravn, Anker Hansen, Ib Jarle Christensen, Thomas Jensen, Bruce Pratt, Jon Askaa, Peter Buhl Jensen, Steen Knudsen, Jens Benn Sørensen

**Affiliations:** 1 Medical Prognosis Institute A/S, Hoersholm, Denmark; 2 Section for Molecular Disease Biology, Faculty of Health and Medical Sciences, University of Copenhagen, Copenhagen, Denmark; 3 Department of Pathology, Copenhagen University Hospital, Rigshospitalet, Denmark; 4 Department of Thoracic Surgery, Copenhagen University Hospital, Rigshospitalet, Copenhagen, Denmark; 5 Oncology Venture Aps, Hoersholm, Denmark; 6 Department of Gastroenterology, Hvidovre Hospital, Hvidovre, Denmark; 7 Department of Oncology, Copenhagen University Hospital, Rigshospitalet, Copenhagen, Denmark; University of South Alabama Mitchell Cancer Institute, UNITED STATES

## Abstract

**Introduction:**

Effective predictive biomarkers for selection of patients benefiting from adjuvant platinum-based chemotherapy in non-small cell lung cancer (NSCLC) are needed. Based on a previously validated methodology, molecular profiles of predicted sensitivity in two patient cohorts are presented.

**Methods:**

The profiles are correlations between *in vitro* sensitivity to cisplatin and vinorelbine and baseline mRNA expression of the 60 cell lines in the National Cancer Institute panel. An applied clinical samples filter focused the profiles to clinically relevant genes. The profiles were tested on 1) snap-frozen tumors from 133 patients with completely resected stage 1B-2 NSCLC randomized to adjuvant cisplatin and vinorelbine (ACV, n = 71) or no adjuvant treatment (OBS, n = 62) and 2) formalin-fixed paraffin-embedded (FFPE) tumors from 95 patients with completely resected stage 1A-3B NSCLC receiving adjuvant cisplatin and vinorelbine.

**Results:**

The combined cisplatin and vinorelbine profiles showed: 1) univariate Hazard Ratio (HR) for sensitive versus resistant of 0.265 (95% CI:0.079–0.889, p = 0.032) in the ACV cohort and a HR of 0.28 in a multivariate model (95% CI:0.08–1.04, p = 0.0573); 2) significant prediction at 3 year survival from surgery in univariate (HR = 0.138 (95% CI:0.035–0.537), p = 0.004) and multivariate analysis (HR = 0.14 (95% CI:0.030–0.6), p = 0.0081). No discrimination was found in the OBS cohort (HR = 1.328, p = 0.60). The cisplatin predictor alone had similar figures with 1) univariate HR of 0.37 (95% CI:0.12–1.15, p = 0.09) in the ACV cohort and 2) univariate HR of 0.14 (95% CI:0.03–0.59, p = 0.0076) to three years. Functional analysis on the cisplatin profile revealed a group of upregulated genes related to RNA splicing as a part of DNA damage repair and apoptosis.

**Conclusions:**

Profiles derived from snap-frozen and FFPE NSCLC tissue were prognostic and predictive in the patients that received cisplatin and vinorelbine but not in the cohort that did not receive adjuvant treatment.

## Introduction

Lung cancer account for 1.59 million deaths annually worldwide and 85% of cases are non-small cell lung cancer (NSCLC) [1]. Approximately 30% of NSCLC patients are eligible for surgery [2,3]. Adjuvant platin-based regimens after surgery are standard care in stage 2A-3A with a robust 5% absolute overall survival (OS) benefit. Conversely, patients with stage 1A do not appear to benefit from the adjuvant chemotherapy and results for stage 1B patients are conflicting [4–6]. Since some of the NSCLC patients receiving cisplatin do not benefit from the treatment, given the controversial results for stage 1B patients, and considering that some of stage 1A patients not receiving cisplatin might actually benefit from it, more effective predictive biomarkers for this treatment appear highly warranted.

Several resistance mechanisms have been identified, which all have major impact on the efficacy of cisplatin [7]. The cytotoxicity of cisplatin is attributed to single strand DNA mono-adducts, intrastrand crosslinks, and interstrand crosslinks. This DNA damage is either repaired by the DNA damage response (DDR), or leads to apoptosis [8]. DDR relevant to the repair of cisplatin-induced DNA damage includes nucleotide excision repair (NER), interstrand crosslink repair (ICLR/FA), mismatch repair (MMR), homologous recombination (HRe) and non-homologous end joining (NHEJ) [9–12]. Cisplatin resistance is shown to be related to DDR proteins such as upregulation of excision repair cross-complementing 1 (ERCC1) as a part of NER and HRe [13], to secondary mutations in breast cancer 1/2, early onset (BRCA1/2) as a part of HRe [14], and to MutS Homologue 2 (MSH2) as a part of MMR and ICL even if conflicting reports exist [15,16]. Other proposed resistance mechanisms to cisplatin are reduced uptake through copper transporters CTR1 and CTR2 and increased efflux through pumps such as ATP7A/ATP7B. All of these have been proposed as prognostic biomarkers and as biomarkers predictive to cisplatin efficacy in the early stages of NSCLC, but possibly due to several competing mechanisms of resistance and efficacy of cisplatin no predictive biomarkers to adjuvant chemotherapy are in use in clinic yet [17].

A more comprehensive, highly multivariate model seems to be required to improve precision in treatment decisions. The model used in the present publication utilizes the full transcriptome as the data source, from which a predictive biomarker is developed. The biomarker system is based on a previously validated method with various drugs in various cancers. The basic hypothesis is that patterns of sensitivity and gene expression can translate into clinical efficacy of each tested drug [18–21]. The National Cancer Institute cell line panel of 60 cell lines (NCI60) is the basis of the drug response predictor (DRP) system and the sensitivity patterns of cisplatin and vinorelbine, respectively, were correlated to gene expression of the same cell lines [22]. This assumes that direct cytotoxic action and other mechanisms of action are not accounted for. The basic profile is then translated in to clinical efficacy of each drug by use of additional gene expression data from 3200 tumors of mixed origin.

From the prospective randomized clinical trial JBR.10 Zhu and colleagues made a dataset of 133 stage 1B-2 NSCLC patients with microarray data publicly available [23]. The 133 patients were randomized to receive either adjuvant chemotherapy with cisplatin and vinorelbine (71 patients) or no adjuvant treatment (62 patients) and mRNA was extracted from snap-frozen tumors [24]. We tested the cisplatin marker, the vinorelbine marker and the combined marker on the dataset.

Using archival formalin-fixed paraffin-embedded (FFPE) tissue the profiles were validated in an independent cohort of stage 1A-3B NSCLC patients (RH-cohort) receiving adjuvant chemotherapy with cisplatin and vinorelbine.

Study objectives were to examine the profiles of cisplatin and vinorelbine individually and combined in the two independent datasets, to evaluate reproducibility of the results, and to interpret any predictive value of the markers to identify benefiters of adjuvant treatment with cisplatin and vinorelbine.

## Materials and methods

### Development of *in vitro* based drug profiles

The model is based on *in vitro* cytotoxicity for each specific drug tested in the NCI60 cell line panel developed prior to the present study by Medical Prognosis Institute [20]. In this project, correlations of cytotoxicity to cisplatin and vinorelbine, respectively, were combined with the transcriptome of the 60 cell lines in the panel. To maintain only clinically relevant genes, mRNA expression was measured in more than 3200 snap-frozen tumor specimens, and only markers present in clinical tumor material were retained. The final signature consists of two sets of genes, features associated with sensitivity and features associated with resistance. We tested both the mRNA and the miRNA transcriptomes in two separate profiles of each drug. The final gene signature is covered by issued (8,445,198) and pending (62/440,883) patents [25]. To interpret any biological meaningful information we did pathway elucidation on the Affymetrix mRNA U133 cisplatin profile. The probes of the cisplatin profile were annotated to corresponding gene names and submitted to g:Profiler for functional interpretation and presented in S1 Doc are a subset of 73 genes that were positively correlated for association to pathways or gene ontologies and had significantly enriched BIOGRID interactions [26].

### JBR.10 dataset

Total RNA from 133 stage 1B-2 NSCLC patients was isolated from snap-frozen tumor samples and hybridized to Affymetrix HG-U133A by Zhu and colleagues. The patients were randomized to receive adjuvant cisplatin and vinorelbine (n = 71; Table 1) or no adjuvant treatment (n = 62) in the JBR.10 randomized clinical trial. This dataset is publicly available on Gene Expression Omnibus as GSE14814 [23].

**Table 1:** Baseline demographics and association in the treated cohorts.

	Study	JBR.10 ACV	JBR.10 OBS	RH
**N**	** **	**71**	**62**	**95**
**Gender**	**Female, n (%)**	24 (33.8)	18 (29.0)	47 (49.5)
	**Male, n (%)**	47 (66.2)	44 (71.0)	48 (50.5)
**Age (yr)**	**median**	62	61	64
	**range**	40–81	35–76	41–78
**DRP association to age (Spearman Rank correlation)**	**p-value**	0.82	0.62	0.46
**Histology**	**AC, n (%)**	39 (54.9)	32 (51.6)	64 (67.4)
	**Other, n (%)**	6 (8.5)	4 (6.5)	15 (15.8)
	**SCC, n (%)**	26 (36.6)	26 (41.9)	16 (16.8)
**DRP association to histology (Kruskal-Wallis)**	**p-value**	0.54	0.83	0.29
**Stage**	**1, n (%)**	39 (54.9)	34 (54.8)	37 (38.9)
	**2, n (%)**	32 (45.1)	28 (45.2)	37 (38.9)
	**3, n (%)**	-		21 (22.1)
**DRP association to stage (Kruskal-Wallis)**	**p-value**	0.54	0.83	0.29
**Adjuvant chemotherapy w/**	**< 2 cycles cisplatin, n**	0	0	26
	**≥** **2 cycles cisplatin, n**	71	0	69
	**< 2 cycles vinorelbine, n**	0	0	25
	**≥** **2 cycles vinorelbine, n**	71	0	70
**Adjuvant radiation (post chemotherapy), n**	** **	0		5
**Surgery**	**Lobectomy, n**	-		76
	**Pneumonectomy, n**	33		13
	**Bilobectomy, n**	-		6
	**Other resection, n**	100		1
**Comorbidities**	PS 0–1*, n	-		55
	**PS** **≥** 2*, n	-		2
	**PS-data missing, n**	-		38
	Diabetes*, n	-		10
	Hypertension*, n	-		36
	COPD or decreased LF*, n	-		40
	Cardiovascular disease*, n	-		23
	**Blood transfusion, n receiving (mean portions received; range of numbers of transfusions)**	-		32 (4.5; 1–19)

Spearman rank correlation or Kruskal-Wallis test was done to assess association with variables and the DRP. In the JBR.10 OBS cohort the histology category Other (n = 4) is significantly different in regards of DRP level. Adenosquamous cell carcinoma is included in the group Other in the analysis. Abbreviations: AC = adenocarcinoma; ACV = adjuvant cisplatin and vinorelbine; COPD = chronic obstructive pulmonary disease; DRP = drug response predictor (profile), the combined cisplatin and vinorelbine predictor; LF = lung function; Other = pleomorphic, spindle cell, high grade mucoepidermoid carcinoma and adenosquamous cell carcinoma; PS = performance status; SCC = squamous cell carcinoma; yr = year.

### RH-cohort

To validate the effect of the mRNA profiles in an independent cohort and to facilitate translation to FFPE material that is by far the most clinically used archival tissue source worldwide [27], FFPE tumor tissue and clinical data from patients treated at Copenhagen University Hospital, Rigshospitalet, during the period 2005–2011 was collected. Ninety-five stage 1A-3B NSCLC patients were included.

Included were patients diagnosed with NSCLC receiving adjuvant treatment with at least one full cycle of cisplatin and vinorelbine following complete macroscopic and microscopic resection of tumor. This cohort of primary NSCLCs comprised the common subtypes of primary NSCLC (Table 1 describing overall features and S1 Table describing detailed histological features of the tumors) [28].

Exclusion criteria were neuroendocrine tumor at time of diagnosis, other cancer within 5 years prior to diagnosis (though basal cell carcinoma of skin, spindle cell carcinoma of skin and carcinoma in situ of cervix were eligible), or breast cancer at any time prior to diagnosis. Further patients known to have metastatic disease (M1) at time of adjuvant treatment were excluded even with complete resection of primary tumor. Patients receiving neoadjuvant chemotherapy were not included in the study. All-comers were 169 patients among whom 95 were included with S1 Fig describing the excluded patients.

The medical history of the eligible patients was obtained retrospectively with at least 3.5 years of clinical follow-up for each patient. Clinical covariates tumor stage, tumor histology and treatment dose were determined from patient records and pathological analysis. Staging and histology were assessed by the same certified pathologist on all samples (ESR) and followed the 7^th^ edition TNM-staging recommended by the International Association for the Study of Lung Cancer (IASLC) [29] and the current WHO Classification of tumors of the lung [28], respectively. When necessary histological type (n = 9) and stage (n = 14) were reclassified. The pathologist further evaluated the percentages of tumor cell content (tumor cell nuclei *vs*. all nuclei in specimen), amount of necrotic tissue, and hemorrhage, as well as possible neuroendocrine features in the examined tissue blocks, as presented in S1 Table.

The microarray data are publicly available on Gene Expression Omnibus as GSE108492.

### Ethics statement

The JBR.10 cohort is previously published and the original research protocol was approved by the institutional review boards at all the institutions, and all patients provided written informed consent [24].

The RH cohort data collection was approval by the Regional Committee on Health Research Ethics for Capital Region Denmark and in accordance with Declaration of Helsinki. Informed consent was not obtained since the research conducted did not have implications on the health or outcome of the enrolled patients which is in accordance with Danish Law and accepted by the Regional Committee on Health Research Ethics for Capital Region Denmark.

### Study design

Cases were selected retrospectively based on inclusion and exclusion criteria as presented above. No stratification was done and there were no matched controls. Primary endpoint was overall survival (OS) and secondary endpoint was disease-specific survival (DSS) (NSCLC-specific survival) evaluated by two observers (IKB, JBS). Only when death was very likely not to be caused by NSCLC, the patients were classified as death of other cause. No additional corrections for co-morbidities were done even though many patients had apparent comorbidities (Table 1). Time is calculated from the date of surgery.

Study sample size was set to approximately 100 patients based on statistical power calculations of 96% with a two-sided alpha of 0.05.

### Laboratory analysis (mRNA and miRNA analysis)

Total RNA was extracted from 5 consecutive 10 μm-thick FFPE sections of tumors resected prior to adjuvant treatment using the RecoverAll™ Total Nucleic Acid Isolation Kit for FFPE samples (Ambion, Austin, TX, USA) according to the manufacturer’s protocol. Total RNA was extracted from 103 tumor samples from 95 patients, amplified and microRNA run on Affymetrix Genechip® miRNA Array 1.0 (Thermo Fisher Scientific, Waltham, MA, USA) and mRNA on Affymetrix Genechip® Human Genome U133 Plus 2.0 Array and Almac Xcel array (Almac Group, Craigavon BT63 5QD, United Kingdom). When more than one tumor sample was obtained from the same lobe, the predictions were compared and the sample with the lowest sensitivity score was kept for evaluation.

Normalization of miRNA and mRNA microarray data was performed in R using Robust Multi-array Average (RMA).

The profile scores were defined as the difference between the averages of the two groups (up- and down-regulated) of features for each of the drugs. The scores were then scaled to cover the range from 0 to 100. One cisplatin-sensitivity score, one vinorelbine score, and one combined score were derived for each patient for each platform.

### Statistics

The association of the drug response predictor (profile) (DRP) marker to the time to the clinical outcomes, disease specific death and death of all causes was assessed using the Cox proportional hazards model on each dataset. Multivariable analysis included age, gender, disease stage and histological type. The DRP marker is scored as a continuous covariate and rescaled so that the hazard ratio (HR) is for a 50-point difference. For each platform, the success criterion is a two-sided p-value of the combined model of less than 0.05.

Model assessment based on martingale residuals demonstrated a significant departure from the proportional hazards assumption in the second dataset (p = 0.013). Therefore, the DRP was entered as a time dependent covariate with a threshold at 3 years. Threshold was defined after the proportional hazards model was rejected but prior to evaluating the method of time dependence.

The two studies were combined using the Cox proportional hazards model with a random effect for study.

Statistical calculations were done using SAS (v9.4, SAS Institute, Cary, N.C., USA) and R (R Development Core Team, Vienna, Austria, http://www.R-project.org).

## Results

### The profiles

The Affymetrix U133 Plus 2.0 mRNA profile for cisplatin consisted of 95 probes corresponding to 83 genes correlated to sensitivity and 110 probes corresponding to 100 genes correlated to resistance. The mRNA vinorelbine profile consisted of 52 upregulated probes and 77 downregulated probes (mRNA). The combined marker was a combination of the cisplatin and vinorelbine profiles at gene level and the values were based on all up- and downregulated genes in both profiles. The mRNA based Affymetrix U133 Plus 2.0 combined marker for cisplatin and vinorelbine is considered the primary profile.

Levels of each predictor and rank correlations between the mRNA predictors of cisplatin, vinorelbine and the combined predictor in the RH-cohort are given in S2 Fig. The mRNA-based cisplatin and the mRNA-based combined cisplatin and vinorelbine profiles correlate with a value of 0.87, p<0.0001. mRNA profiling on Almac Xcel assay was almost indistinguishable from the Affymetrix mRNA U133 Plus 2.0 (correlation coefficient = 0.96 for cisplatin profile), hence we report the Affymetrix mRNA U133 Plus 2.0 results in the following.

The miRNA for cisplatin alone and the combined miRNA cisplatin and vinorelbine correlate poorly to the mRNA markers (p = 0.26 and p = 0.74).

### Baseline demographics and association

Baseline demographic features of the JBR.10 subpopulation are presented in the original publication by Zhu and colleagues and in Table 1 [23].

Baseline clinical and histological characteristics of the 95 consecutive stage 1A-3B NSCLC patients in the RH-cohort are presented in Table 1. The median time of observation was 76.6 months (reverse Kaplan-Meier method). 43 patients had died with 36 deaths attributed to NSCLC.

The RH-cohort appeared representative of a NSCLC population even with some differences to the JBR.10 cohort [28]. While JBR.10 cohort had 55% adenocarcinoma there were 67% in the RH-cohort and adenocarcinomas had poorer survival than squamous cell carcinomas (p = 0.03). As expected disease stage was a singular prognostic marker (p = 0.02) with outliers in stage 1A that represent too few individuals to be accounted for.

The two cohorts are in general comparable with respect to gender, age and treatments received. There are some differences in regards of the parameters histology and stage. In the RH-cohort, all patients that had radical surgery and ACV from 2005–2011 at Rigshospitalet, Copenhagen, Denmark, were enrolled. Hence included were some outliers in regards of stage (1A and 3B). JBR.10 included only stage 1B-2.

Table 1 shows that there is no association between the combined vinorelbine-cisplatin marker and clinical covariates gender, histology, stage, or age in either datasets (p-values ranges from 0.29–0.83).

### Profiles and prognosis

#### JBR.10-cohort

**Fig 1:**
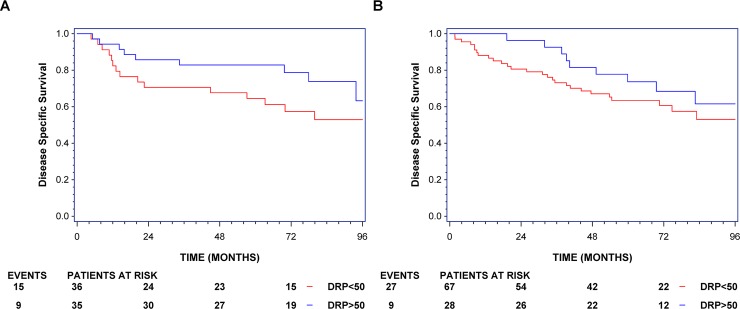
Kaplan-Meier curves of the cohorts receiving adjuvant cisplatin and vinorelbine, disease-specific survival. The curves show the cohort receiving adjuvant chemotherapy (ACV) in JBR.10 (1A) divided by a score of 50 and of the RH-cohort also receiving ACV (1B) divided by a score of 50. Underneath each curve is a description of events and patients at risk at different time points. Red: Combined cisplatin and vinorelbine score > 50, predicted high-likelihood responders to ACV; black: Combined cisplatin and vinorelbine score ≤ 50, predicted low-likelihood responders to ACV.

Kaplan-Meier estimates of disease-specific survival (DSS) of the cohort treated with ACV divided by a score of the combined markers of 50 is presented in Fig 1A. The combined cisplatin and vinorelbine marker profiles in the first cohort scored as a continuous covariate showed a Hazard Ratio (HR) = 0.265 (95% CI:0.079–0.889, p = 0.032) in the ACV cohort (sensitive versus resistant), as shown in Table 2. Similar effect sizes were seen when dichotomizing the profile by the median, HR = 0.52 (95% CI:0.228–1.190, p = 0.12). A multivariate model adjusted for stage demonstrated significance for ACV (HR = 0.284 (95% CI:0.086–0.944), p = 0.040) but in a model with stage, sex, 10-year age difference and histology the score only did show a trend (HR = 0.28 (95% CI: 0.08–1.04), p = 0.0573, Table 2). Neither univariate nor multivariate models of the combined markers were statistically significant with overall survival as endpoint (Table 2).

**Table 2:** Uni- and multivariate model per study, endpoint disease-specific survival and overall survival.

				Endpoint					
				Disease-Specific Survival			Overall Survival		
				Hazard Ratio	95% Confidence Limit for Hazard Ratio	P-value	Hazard Ratio	95% Confidence Limit for Hazard Ratio	P-value
**Model**	**Cohort**	**Parameter**	**Level**						
**1: Univariate**	**1**	**DRP 50-point difference**	** **	0.26	(0.08–0.89)	**0.032**	0.41	(0.15–1.15)	0.09
	**2**	**DRP 50- point difference**	** **	0.38	(0.15–0.98)	**0.045**	0.61	(0.26–1.42)	0.25
**2: Univariate Time Dependent**	**1**	**DRP** **≤** **3 years**	** **	0.26	(0.06–1.16)	0.08	0.26	(0.07–1.06)	0.06
		**DRP > 3 years**	** **	0.27	(0.03–2.21)	0.22	0.75	(0.16–3.40)	0.70
	**2**	**DRP** **≤** **3 years**	** **	0.14	(0.04–0.54)	**0.0042**	0.17	(0.05–0.64)	**0.0082**
		**DRP > 3 years**	** **	1.08	(0.28–4.15)	0.91	1.68	(0.53–5.30)	0.38
**3: Multivariate model**	**1**	**Gender**	**Female**	0.91	(0.33–2.51)	0.86	0.75	(0.31–1.80)	0.52
		**Age 10-year difference**		1.65	(0.98–2.79)	0.059	1.62	(1.02–2.59)	**0.042**
		**DRP 50-point difference**		0.28	(0.08–1.04)	0.057	0.45	(0.16–1.30)	0.14
		**Histology (vs. AC)**	**Other**	1.46	(0.37–5.68)	0.59	0.88	(0.24–3.20)	0.84
			**SCC**	0.40	(0.14–1.16)	0.09	0.30	(0.12–0.79)	**0.014**
		**Stage (vs. 1)**	**2**	1.73	(0.74–4.01)	0.20	1.21	(0.58–2.53)	0.62
	**2**	**Gender**	**Female**	0.87	(0.42–1.80)	0.71	0.93	(0.48–1.82)	0.84
		**Age 10-year difference**		1.50	(0.96–2.35)	0.08	1.55	(1.01–2.37)	**0.044**
		**DRP 50-point difference**		0.41	(0.14–1.21)	0.11	0.67	(0.25–1.78)	0.42
		**Histology (vs. AC)**	**Other**	0.50	(0.18–1.39)	0.18	0.50	(0.19–1.27)	0.14
			**SCC**	0.21	(0.05–0.93)	**0.040**	0.35	(0.12–1.08)	0.07
		**Stage (vs. 1)**	**2**	2.45	(1.04–5.77)	**0.040**	2.93	(1.32–6.47)	**0.0080**
			**3**	2.58	(1.06–6.29)	**0.037**	3.44	(1.47–8.05)	**0.0043**
**4: Multivariate Time-dependent analysis per study**	**1**	**Gender**	**Female**	0.91	(0.33–2.50)	0.86	0.76	(0.32–1.84)	0.55
		**Age 10-year difference**		1.66	(0.98–2.80)	0.058	1.62	(1.02–2.58)	**0.042**
		**Histology (vs. AC)**	**Other**	1.46	(0.37–5.67)	0.59	0.89	(0.24–3.28)	0.86
			**SCC**	0.40	(0.14–1.15)	0.09	0.31	(0.12–0.82)	**0.018**
		**Stage (vs. 1)**	**2**	1.73	(0.75–4.02)	0.20	1.20	(0.57–2.52)	0.64
		**DRP** **≤** **3 years**	** **	0.31	(0.07–1.48)	0.14	0.32	(0.08–1.35)	0.12
		**DRP > 3 years**	** **	0.22	(0.02–2.29)	0.21	0.70	(0.14–3.41)	0.66
	**2**	**Gender**	**Female**	0.92	(0.44–1.90)	0.81	0.99	(0.50–1.94)	0.97
		**Age 10-year difference**		1.50	(0.95–2.37)	0.08	1.53	(1.00–2.36)	0.052
		**Histology (vs. AC)**	**Other**	0.52	(0.19–1.44)	0.21	0.53	(0.21–1.35)	0.18
			**SCC**	0.21	(0.05–0.94)	**0.042**	0.36	(0.12–1.11)	0.07
		**Stage (vs. 1)**	**2**	2.48	(1.07–5.74)	**0.035**	2.98	(1.36–6.49)	**0.0061**
			**3**	2.61	(1.07–6.35)	**0.034**	3.49	(1.50–8.13)	**0.0038**
		**DRP** **≤** **3 years**		0.15	(0.03–0.63)	**0.010**	0.17	(0.04–0.72)	**0.016**
		**DRP > 3 years**		1.37	(0.29–6.42)	0.69	2.24	(0.60–8.37)	0.23

Part 1 represent a univariate model per cohort, part 3 represent a multivariate model per cohort. Parts 2 and 4 represent the time-dependent analysis conducted on each cohort in a uni- and multivariate model respectively. All hazard ratios for DRP are based on a continuous score with a 50-point difference. Cohort 1 refers to the JBR.10 cohort treated with cisplatin and vinorelbine and cohort 2 refers to the RH-cohort. Adenosquamous cell carcinoma is included in the group Other in the analysis. Abbreviations: AC = adenocarcinoma; ACV = adjuvant cisplatin and vinorelbine; DRP = drug response predictor (profile), the combined cisplatin and vinorelbine predictor; Other = pleomorphic, spindle cell, high grade mucoepidermoid carcinoma and adenosquamous cell carcinoma; SCC = squamous cell carcinoma.

**Fig 2:**
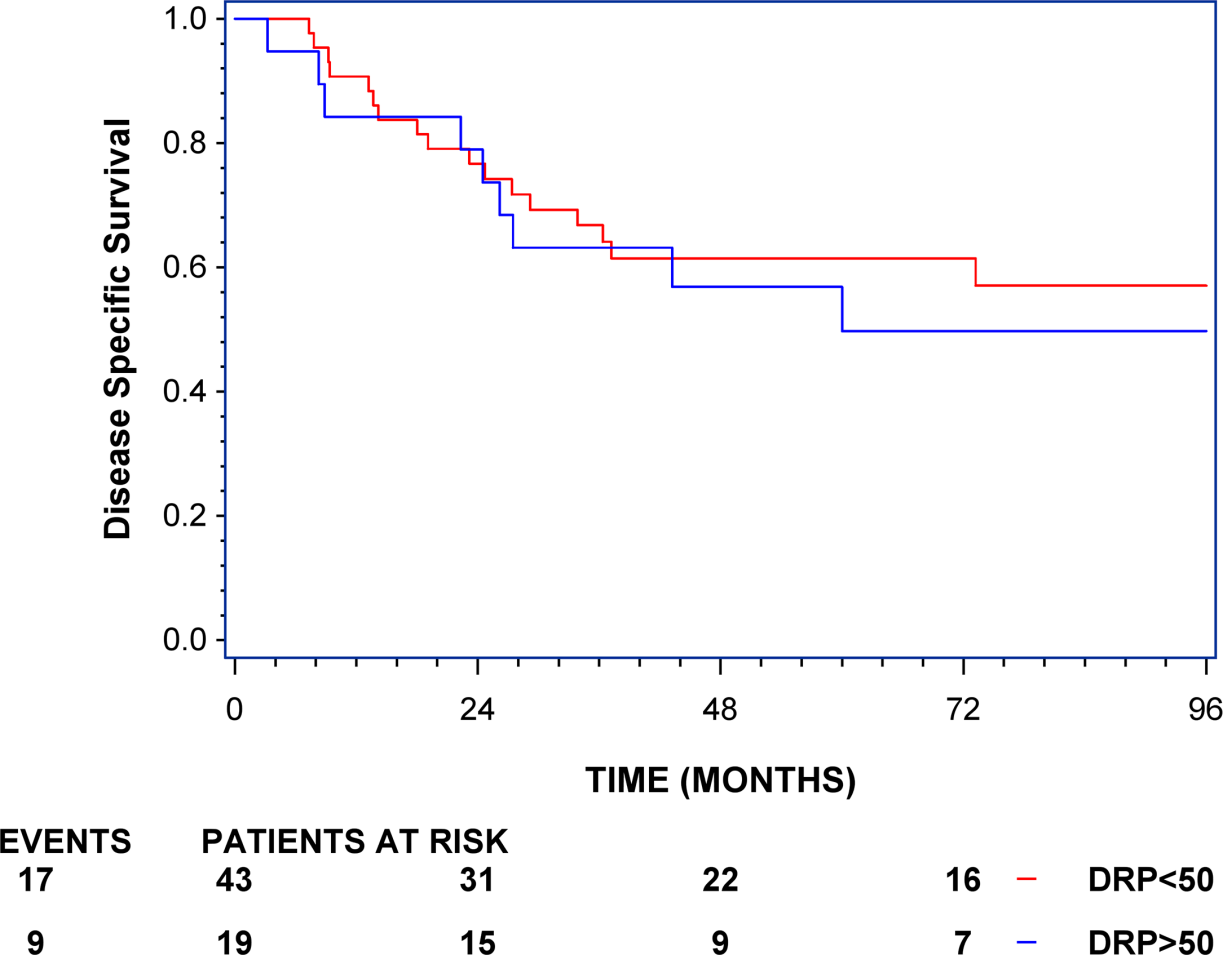
Kaplan-Meier curve of observational cohort (OBS) JBR.10, disease-specific survival. The curves show the observational cohort from JBR.10 divided by a score of 50. Underneath the curve is a description of events and patients at risk at different time points. Black: Combined cisplatin and vinorelbine score > 50, predicted high-likelihood responders to ACV; red: Combined cisplatin and vinorelbine score ≤ 50, predicted low-likelihood responders to ACV.

In the control arm that had no adjuvant treatment (OBS) the Kaplan-Meier estimates of DSS divided by a score of the combined markers of 50 is presented in Fig 2. No significant discrimination in the OBS cohort was seen when scoring as a continuous covariate (HR = 1.328 (95% CI:0.46–3.835), p = 0.60). A multivariate model adjusted for stage confirmed the hazard ratios above and was also not statistically significant (HR = 1.702 (95% CI: 0.575–5.036), p = 0.34).

#### RH-cohort

Kaplan-Meier estimates of DSS of the RH-cohort treated with ACV divided by a score of 50 is presented in Fig 1B.

Due to significant deviation from the proportional hazards assumption in the RH-cohort a time-dependent analysis was warranted. The combined cisplatin and vinorelbine marker profile resulted in a significant prediction for up to 3 years from surgery when using a time-dependent Cox model for a difference of 50 points (Table 2). The HR for 3 years OS was 0.17 (95% CI: 0.05–0.64, p = 0.008) and similarly for DSS the HR was 0.138 (95% CI:0.035–0.537), p = 0.004) scored as a continuous covariate. HR for 5 years DSS was 0.35 (95% CI:0.13–1.00, p = 0.050, univariate).

In the time-dependent multivariate model adjusting for stage, gender, 10-year difference of age and histology with a 50-point difference in the score, the HR for 3 years OS was 0.17 (95% CI: 0.04–0.68, p = 0.013) and for DSS HR was 0.14 (95% CI: 0.03–0.60, p = 0.008), as shown in Table 2. In the 5-year multivariate model for DSS HR was 0.40 (95% CI:0.12–1.28, p = 0.12).

A multivariate model with endpoint DSS adjusting for stage only showed that the predictor remained significant (HR = 0.123 (95% CI:0.030–0.512), p = 0.004).

From three years and beyond, all the estimated HRs were above 1 in the RH-cohort and none were statistically significant.

Additional multivariable analyses using the cisplatin DRP alone revealed similar results as the combined predictor with OS (HR 0.37 (95% CI: 0.19–0.70), p = 0.0024, S2 Table) and DSS (HR 0.37 (95%CI: 0.20–0.72) p = 0.0030, S2 Table) to three years and no effect in the group from three years and beyond.

We also tested a microRNA cisplatin DRP predictor with OS (S3 Table) that revealed no statistically significant prediction at any time and we did not continue with any analysis on the miRNA predictor.

### Pooled analysis of the mRNA data

A pooled analysis of the two treated cohorts with the combined marker with a 50-point difference for endpoint DSS resulted in a significant prediction (HR = 0.187, (95% CI 0.069–0.508), p = 0.001) up to 3 years from surgery using a random effects model. The entire length of the studies univariate was also significant (HR = 0.34 (95% CI 0.17–0.71), p = 0.0042).

The DRP predicted significantly in the multivariate model with 50-point difference and time-dependent analysis at three years (HR = 0.21 (95% CI 0.07–0.60), p = 0.0036), shown in Table 3. When assessing the effect in the pooled studies from three years and beyond, the effect was not significant (HR = 0.76 (95% CI 0.24–2.45), p = 0.6510). When assessing the effect in the entire length of the studies in a multivariate model of the pooled studies and both markers the estimates were significant (HR 0.35 (95% CI 0.16–0.78), p = 0.0096).

**Table 3:** Pooled cohorts, multivariate time-dependent model, endpoint disease-specific survival.

			Hazard Ratio	95% Confidence Limit for Hazard Ratio	P-value
**Model**	**Parameter**	**Level**			** **
**Multivariate Time-dependent**	**Gender**	**Female**	0.90	(0.51–1.60)	0.72
	**Age 10-year difference**	** **	1.57	(1.11–2.21)	**0.010**
	**Histology (vs. AC)**	**Other**	0.74	(0.33–1.62)	0.45
		**SCC**	0.34	(0.15–0.75)	**0.0080**
	**Stage (vs. 1)**	**2**	1.94	(1.09–3.47)	**0.025**
		**3**	2.24	(1.05–4.78)	**0.037**
	**DRP** **≤** **3 years**	** **	0.21	(0.07–0.60)	**0.0036**
	**DRP > 3 years**	** **	0.76	(0.24–2.45)	0.6510

The table shows the time-dependent analysis conducted on the pooled cohorts in a multivariate model. Hazard ratios for DRP are based on a continuous score with a 50-point difference. Adenosquamous cell carcinoma is included in the group Other in the analysis. Abbreviations: AC = adenocarcinoma; ACV = adjuvant cisplatin and vinorelbine; DRP = drug response predictor (profile), the combined cisplatin and vinorelbine predictor; Other = pleomorphic, spindle cell, high grade mucoepidermoid carcinoma and adenosquamous cell carcinoma; SCC = squamous carcinoma.

### A model with variable thresholds combined with prognostic features

**Fig 3:**
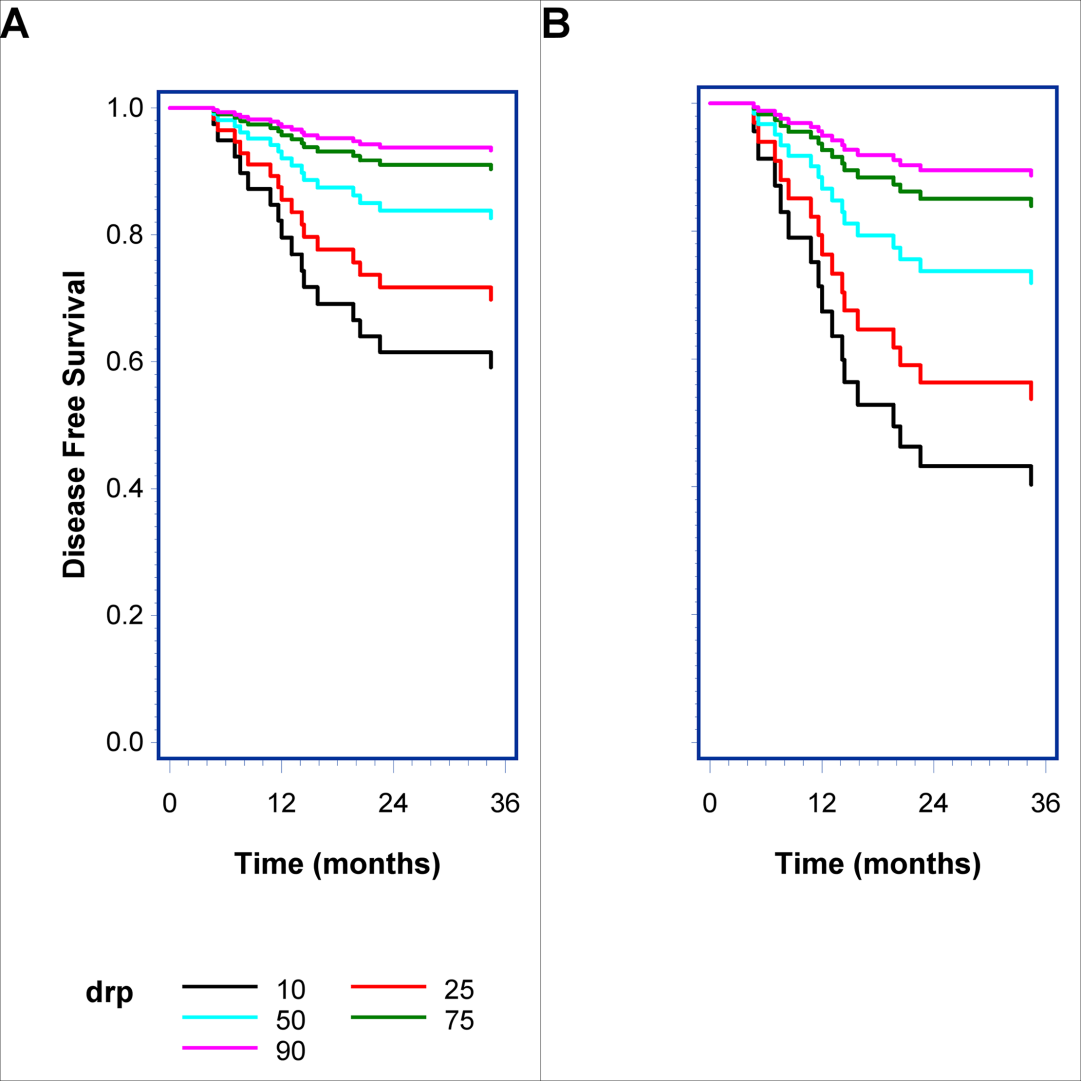
Expected disease-specific survival based on a multivariable model in the ACV cohort of JBR.10. Curves shown for expected disease-specific survival based on the multivariate time-dependent model from JBR.10 with values of the combined profiles (DRP) of 10, 25, 50, 75 and 90 for a model including gender male, age 62 years, histology adenocarcinoma and stage 1 (A) and stage 2 (B).

To search for a clinical feasible cut-off, we plotted the expected survival in the ACV cohort of JBR.10 in Fig 3 based on the multivariate model in Table 2. The plot shows expected survival curves for values of the combined profiles at value levels 10, 25, 50, 75, 90 in a linear model with gender set as male, age set as 62 years (median in that cohort), histology as adenocarcinoma and stage as 1 (Fig 3A) and 2 (Fig 3B), respectively.

### Analysis of the cisplatin profile genes’ functional interrelationship

The genes of the cisplatin profile genes were submitted to g:Profiler and of the 83 genes in the sensitivity profile, 38 genes had significantly enriched BIOGRID interactions and were thus biologically correlated. Similarly, of the 100 genes in the resistance profile, 35 were had enriched BIOGRID interactions (S1 Doc) [26].

Of those genes some revealed patterns of interest in relation to cisplatin and known biochemistry related to cisplatin efficacy and resistance and the following section describes such findings in the profiles.

The mechanism of action of cisplatin relies on DNA damage which ultimately gives rise to apoptosis. Conversely, the DNA damage response (DDR) of cells may impair the efficacy of the drug by removing and repairing the cisplatin-mediated DNA lesions. Further alternative splicing of some proteins can switch them from anti-apoptotic to pro-apoptotic function as with the example of BCL-X_L_ (also present in the profile) [30,31].

None of the canonical DDR proteins appear in the profiles. However, several proteins, originally associated with RNA editing including alternative splicing, such as *SFPQ*, *SYNCRIP*, *DICER1*, *SRSF7*, *snRNP70*, *QKI* do appear in the sensitivity profile. Recent research has shown that these proteins also participate in repair of double strand DNA breaks, which can be caused by cisplatin [32–36]. Moreover, *IFI16* is a BRCA1 binding partner and interacts with p53 and is upregulated in the sensitivity profile [37].

Both CD93 and Moesin (*MSN*) are involved in apoptosis and retained in the sensitivity profile in line with theory [38] and *ANP32E* that removes histone H2A.Z, a splice variant of a histone, is involved in several cancer types [39].

In the resistance profile *BCL2L1* coding for the protein BCL-X_L_ is a predictor of cisplatin resistance in ovarian cancer [40,41]. Glutathione peroxidase 2 (*GPX2*) is upregulated by *NR2F2* which is also in the resistance profile [42]. Loss of keratin 8 and 18 (*KRT8*, *KRT18*), both present in the resistance profile, has been shown in epithelial cancer cells to increase cisplatin sensitivity and cell migration and are known factors in epithelial-mesenchymal transition [43].

Overall, we found that potential relevant pathways in prediction of cisplatin efficacy are genes that relate to RNA editing possibly as playing a role in repairing DNA double-strand breaks, but also there are some implications of a connection to both apoptosis in the sensitivity profile and epidermal-mesenchymal transition in the resistance profile.

## Discussion

With the emergence of new effective therapies in advanced stages of NSCLC personalizing treatment by biomarker-guidance has become more relevant. This is also relevant in the adjuvant setting as alternative treatments to cisplatin may appear in the nearer future.

In the current study, two sets of up-and downregulated genes reflecting sensitivity/ resistance to cisplatin and vinorelbine, respectively, were tested in two independent cohorts of NSCLC patients in the adjuvant setting. This method of identification of predictive biomarker profiles has been applied previously in various cancers with various drugs, *e*.*g*. adjuvant 5FU in colorectal cancer and fulvestrant in breast cancer [20,21].

The basic hypothesis of the biomarker system is that *in vitro* sensitivity can translate in to clinical utility through an algorithm based on more than 3200 tumors to sort away genes and pathways only expressed *in vitro*. The system has also been externally validated by statisticians at MD Anderson that received blinded predictions and evaluated the accuracy to outcome [18].

In this study, the most important results are the consistent effects of the combined markers of cisplatin and vinorelbine through two independent treated cohorts. Further, the cisplatin marker behaves consistently alone and combined with the vinorelbine marker in the two cohorts. The fact that the markers did not predict an effect in the population that had only surgery emphasizes that the markers are predictive of adjuvant cisplatin and vinorelbine efficacy and not merely prognostic.

Focus should also be put on the stability of the marker system through NSCLC tissue state, since the markers were tested initially on mRNA from snap-frozen tissue in the JBR.10 cohort and then validated on mRNA from FFPE tissue in the RH-cohort. In this respect, FFPE tissue notoriously contains mRNA of poorer quality than the one obtainable from snap-frozen tissue, but on the other hand it is by far the most used material for diagnostic and predictive purposes in the clinical setting [27]. Thus, the robustness of the markers’ validation on FFPE tumor tissue indeed highlights the potential clinical utility of the DRP.

With promising results from ovarian cancer we also tested a microRNA predictor of cisplatin on the RH-cohort [44], however this did not result in any relevant prediction in the current study.

In the RH-cohort we conducted a time-dependent analysis with a cut-off of 3 years as the primary analysis. Follow-up time was at least 3.5 years and therefore a cut-off of 3 years seems reasonable. Most studies consider 5 year survival time and hence we presented those as well. The effect of the biomarker in the RH-cohort is by far the largest before 3 years. The effect weakens with time and at 5 years is just on the 0.05 cut-off of p-value, univariately. Beyond a 5 year cut-off the effect is not visible neither in OS nor DSS. The expectation must though be a pronounced importance of the biomarkers in the beginning of the treatment course. This could be reflecting the increased lung cancer-independent mortality described in lung cancer cohorts as the patients in the RH-cohort are multimorbid patients (Table 1) [45]. Smoking status was not accounted for in the patient records and smoking could be a significant variable influencing outcomes. One could hypothesize that the failure of the DRP to predict a benefit beyond 3 years is related to the development of independent primary tumors due to the mutagenic field effect of tobacco smoke exposure.

Multigene setups are appealing when no other known effective single-hit biomarkers exist, which is the case for most anticancer drugs. Similar to our group, other groups have developed multigene signatures for prediction of treatment efficacy in NSCLC, but still none have entered into clinical practice [23,46–48]. However, we believe multigene profiles will be included in future decision processes as seen *e*.*g*. in the prognostication of breast cancer guiding patients to adjuvant therapy [49].

Furthermore, the DRP system could be regarded as hypothesis-generating, as genes and pathways involved in cisplatin efficacy and resistance were not all identified previously.

In the cisplatin profile, genes related to RNA editing were abundant and with recent studies pointing to a role in repairing DNA double-strand breaks. Furthermore, there are some implications of a connection to both apoptosis in the sensitivity profile and epithelial-mesenchymal transition in the resistance profile, whereas neither ERCC1, BRCA1 nor MSH2 appeared in the profiles. This could point to new paths of research on cisplatin efficacy and resistance.

### A combined model

Based on simple marker threshold values from the current study, a prospective phase 2 biomarker-guided study with the cisplatin marker as a companion diagnostic has started. It is enrolling heavily-pretreated breast cancer patients above a specific score to be treated with liposomal cisplatin [50,51]. But could further elaboration on the model for instance combined with known prognostic factors refine the system? We propose a model as seen in Fig 3 with a continuous score combined with known prognostic factors such as stage and histology. The predicted model in Fig 3 could work as a tool to open a transparent discussion with the patient in an evaluation of expected benefit against toxicity and comorbidity. This could enroll all stage 1 patients with high profile levels (e.g. cutoff above 30%) to treatment with cisplatin. Or it could eventually exclude patients that are not expected to benefit such as frail stage 2 patients in the lower quarter level of the profile. This system could support the open conversation between physician and patient of benefits versus toxicities to treatment.

### Conclusions

Multigene biomarkers of cisplatin and vinorelbine robustly identified benefiters of treatment with cisplatin and vinorelbine through two independent cohorts and in both snap-frozen and FFPE tissue, which emphasizes their potential clinical applicability. Since the markers showed no prognostic ability in the observational cohort, they appear to be predicting actual treatment benefit.

Biomarkers of drug efficacy are very much called for with anticipated alternate treatment options to cisplatin in all stages of NSCLC. And the current biomarker system could already be useful today as a decision-making tool in stage 1A and 1B where there are uncertainties regarding clinical benefit of cisplatin. Similarly, we see this as a decision making tool with frail patients in later stages to support any treatment decision.

## Supporting information

S1 Doc Sensitivity and resistance genes in the cisplatin profile from the g:profiles pathway analysis. (DOCX)

S1 Fig Overview of excluded patients in the RH-cohort. All comers were 169 patients. 74 patients were excluded due to various reasons as described in the figure.
(TIFF)

S2 Fig Levels and correlations between mRNA profiles in the RH-cohort. Presented on the diagonal in the figure is the distribution of the Affymetrix U133 Plus 2.0 mRNA (normalized) cisplatin score, vinorelbine score and computed combined score in the RH-cohort. The panels in the lower left part of the figure show scatter plots of two scores at a time, while the numbers in the upper right panels show the corresponding Spearman correlation coefficients and the p-values for the correlation coefficient.
(TIFF)

S1 Table Histological features of tumors from the RH-cohort. Abbreviations: AC = adenocarcinoma, ACV = adjuvant chemotherapy, ASCC = adenosquamous cell carcinoma, SCC = squamous cell carcinoma.
(DOCX)

S2 Table U133 CIS predictor, endpoint DSS and OS. Part 1 represent a univariate model per cohort, part 3 represent a multivariate model per cohort. Parts 2 and 4 represent the time-dependent analysis conducted on each cohort in a uni- and multivariate model respectively. All hazard ratios for DRP are based on a continuous score with a 50-point difference. Cohort 1 refers to the JBR.10 cohort treated with cisplatin and vinorelbine and cohort 2 refers to the RH-cohort. Adenosquamous cell carcinoma is included in the group Other in the analysis. Abbreviations: AC = adenocarcinoma; ACV = adjuvant cisplatin and vinorelbine; DRP = drug response predictor (profile), the combined cisplatin and vinorelbine predictor; Other = pleomorphic, spindle cell, high grade mucoepidermoid carcinoma and adenosquamous cell carcinoma; SCC = squamous carcinoma.
(DOCX)

S3 Table Mir CIS predictor, endpoint OS. Multivariate analysis of the miRNA cisplatin predictor in a time-dependent model with a cut-off of 3 years. The predictor is scored as a continuous variable and the hazard ratio estimates are for a 50-point difference. Adenosquamous cell carcinoma is included in the group Other in the analysis. Abbreviations: AC = adenocarcinoma; Other = pleomorphic, spindle cell, high grade mucoepidermoid carcinoma and adenosquamous cell carcinomas; SCC = squamous carcinoma.
(DOCX)
