# Supplementary material for: Molecular prediction of adjuvant cisplatin efficacy in Non-Small Cell Lung Cancer (NSCLC)—validation in two independent cohorts
Source: PLoS One. 2018 Mar 22;13(3):e0194609. doi: 10.1371/journal.pone.0194609 (PMC5864030; doi:10.1371/journal.pone.0194609)
Supplement: S1 Doc — (DOCX) [file pone.0194609.s001.docx]

# **S1 Doc. Sensitivity and resistance genes in the cisplatin profile from the g:profiles analysis.**

**Sensitivity genes in the cisplatin profile from the g:profiles analysis**

Outcome from the g:profiles pathway analysis are 38 genes: ARHGAP15 = Rho GTPase activating protein 15, ARHGEF6 = Rac/Cdc42 guanine nucleotide exchange factor 6, BNIP3 = BCL2/adenovirus E1B 19kDa interacting protein 3, CBLB = Cbl protooncogene B, E3 ubiquitin protein ligase, CD47 = Cd47, CD93 = CD93, CORO1A = coronin, actin binding protein, 1A, CORO1B = coronin, actin binding protein, 1B, DICER1 = Dicer1, ribonuclease type III, EIF4A1 = eukaryotic translation initiation factor 4A1, EVL = Enah/Vasp-like, FAM46A = Family with sequence similarity 46, member A, FLI1 = Fli-1 protooncogene, ETS transcription factor, FMNL1 = formin like 1, FNBP1 = Formin binding protein 1, FTL = ferritin light polypeptide, GLYR1 = Glyoxylate reductase 1 homolog (Aradidopsis), HCLS1 = hematopoietic cell-specific Lyn substrate 1, IFI16 = interferon gamma inducible protein 16, ITGA4 = Integrin Alpha 4, LCP1 = Lymphocyte cytosolic protein 1 (L-plastin), MBNL1 = musclebind like splicing regulator 1, MFF = mitochondrial fission factor, MSN = moesin, PDE4DIP = phosphodiesterase 4D interacting protein, PLEKHO1 = pleckstrin homology domain containing family O member 1, PTPN7 = protein tyrosine phosphatase non-receptor 7, PWP1 = pwp1 homologue, QKI = QKI, KH domain containing, RNA binding, RAP1B = RAP1B member of Ras oncogene family, SFPQ = splicing factor prolinerich, SNRNP70 = snRibonuclear subunit 70kDa, SRRM1 = serine/arginine repetitive matrix 1, SRSF7 = serine/arginine rich splicing factor 7, SYNCRIP = synaptotagmin binding, cytoplasmatic RNA interacting protein, TRAF3 = TNF receptor-associated factor 3, TRAF3IP3 = TRAF3 interacting protein 3, VIM = vimentin.

**Resistance genes in the cisplatin profile from the g:profiles analysis.**

Outcome from the g:profiles pathway analysis are 35 genes: AREG = amphiregulin, BCL2L1 = BCL2 like 1, CTBP2 = c-terminal binding protein 2, DSG2 = desmoglein 2, EHD1 = EH domain containing 1, EPCAM = epithelial cell adhesion molecule, FBP1 = fructose-bisphosphatase 1, FCGRT = Fc fragment of IgG receptor and transporter, FGFR4 = fibroblast growth factor receptor 4, GCNT3 = glucosaminyl (N-acetyl) transferase 3, mucin type, ITGB4 = integrin subunit beta 4, JUP = junction plakoglobin, KRT18 = keratin 18 type 1, KRT8 = keratin 8 pseudogene 3, LAD1 = ladinin 1, LCN2 = lipocalin 2, LGALS3 = galectin 3, LRP5 = LDL receptor related protein 5, LSR = lipolysis stimulated lipoprotein receptor, MCCC2 = methylcrotonoyl-CoA carboxylase 2, MST1R = macrophage stimulating 1 receptor, MUC13 = mucin 13, cell surface associated, NR2F2 = nuclear receptor subfamily 2 group F member 2, NT5E = 5’-nucleotidase ecto, PCK2 = phosphoenolpyruvate carboxykinase 2, mitochondrial, PPARG = peroxisome proliferator activated receptor gamma, RAB11FIB1 = RAB11 family interacting protein 1, S100A11 = S100 calcium binding protein A11, SFN = stratifin, SH2D3A = SH2 domain containing 3A, SLC3A2 = solute carrier family 3 member 2, SORL1 = sortilin related receptor 1, SOX9 = SRY-box 9, TCF7L2 = transcription factor 7 like 2, TPD52L2 = tumor protein D52-like 2.
