# Supplementary figures and images for: Molecular prediction of adjuvant cisplatin efficacy in Non-Small Cell Lung Cancer (NSCLC)—validation in two independent cohorts
Source: PLoS One. 2018 Mar 22;13(3):e0194609. doi: 10.1371/journal.pone.0194609 (PMC5864030; doi:10.1371/journal.pone.0194609)

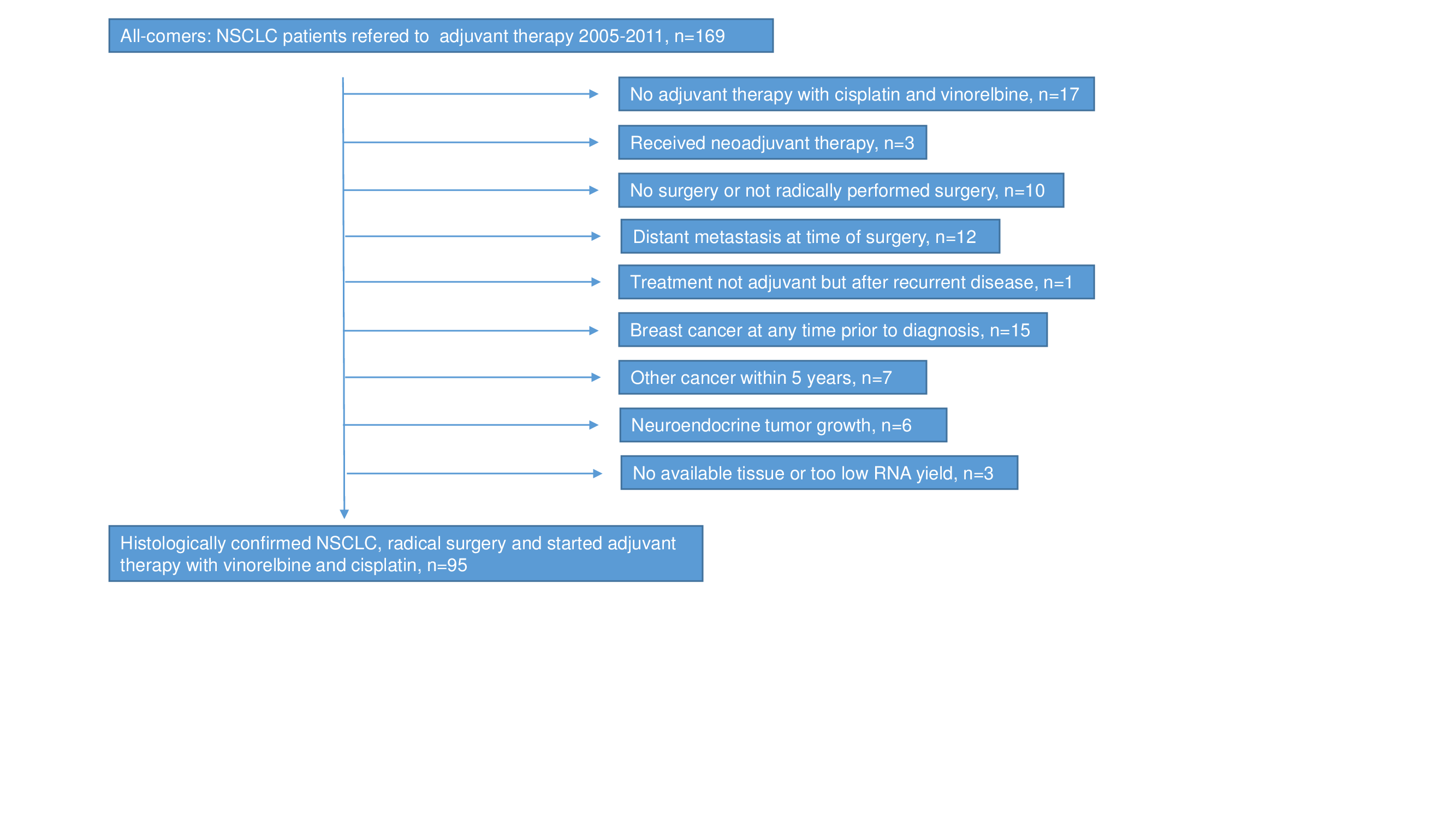

Supplement: S1 Fig — All comers were 169 patients. 74 patients were excluded due to various reasons as described in the figure. (TIFF) [file pone.0194609.s002.tiff]

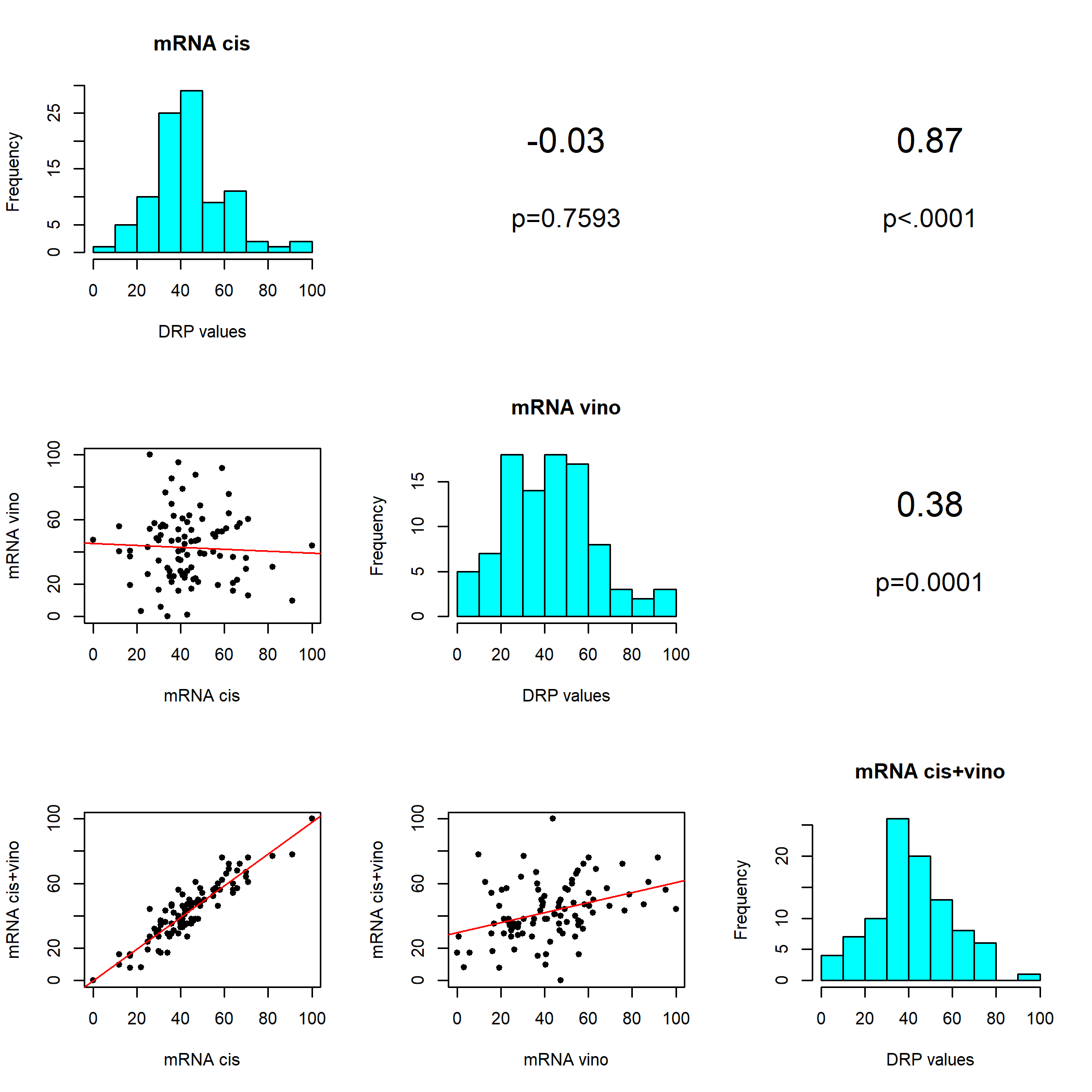

Supplement: S2 Fig — Presented on the diagonal in the figure is the distribution of the Affymetrix U133 Plus 2.0 mRNA (normalized) cisplatin score, vinorelbine score and computed combined score in the RH-cohort. The panels in the lower left part of the figure show scatter plots of two scores at a time, while the numbers in the upper right panels show the corresponding Spearman correlation coefficients and the p-values for the correlation coefficient. (TIFF) [file pone.0194609.s003.tiff]
