# Supplementary material for: Molecular prediction of adjuvant cisplatin efficacy in Non-Small Cell Lung Cancer (NSCLC)—validation in two independent cohorts
Source: PLoS One. 2018 Mar 22;13(3):e0194609. doi: 10.1371/journal.pone.0194609 (PMC5864030; doi:10.1371/journal.pone.0194609)
Supplement: S1 Table — Abbreviations: AC = adenocarcinoma, ACV = adjuvant chemotherapy, ASCC = adenosquamous cell carcinoma, SCC = squamous cell carcinoma. (DOCX) [file pone.0194609.s004.docx]

**S1 Table. Histological features of tumors from the RH-cohort.**

|  |  | **RH-cohort (n)** |
| --- | --- | --- |
| **Histology subtyping** | **AC, solid** | 33 |
|  | **AC, acinar** | 22 |
|  | **AC, papillary** | 5 |
|  | **AC, micropapillary** | 1 |
|  | **AC, lepidic** | 2 |
|  | **AC, mucinous** | 1 |
|  | **SCC** | 16 |
|  | **Other, ASCC** | 8 |
|  | **Other, pleomorphic** | 5 |
|  | **Other, spindle cell carcinoma** | 1 |
|  | **Other, high grade mucoepidermoid carcinoma** | 1 |
| **Differentiation** | **Low** | 49 |
|  | **Intermediate** | 37 |
|  | **High** | 7 |
|  | **Missing** | 2 |
| **Stage** | **1A** | 4 |
|  | **1B** | 33 |
|  | **2A** | 25 |
|  | **2B** | 12 |
|  | **3A** | 19 |
|  | **3B** | 2 |
| **Tumor cell content** | **75-100 %** | 10 |
|  | **50-75 %** | 43 |
|  | **25-50 %** | 23 |
|  | **5-25 %** | 18 |
|  | **<5 %** | 0 |
| **Necrosis** | **None** | 26 |
|  | **5-10 %** | 29 |
|  | **15-20 %** | 12 |
|  | **25-30 %** | 11 |
|  | **35-40 %** | 7 |
|  | **45-50 %** | 9 |
|  | **90 %** | 1 |
| **Hemorrhage** | **None** | 78 |
|  | **5-10 %** | 12 |
|  | **15-20 %** | 3 |
|  | **25-30 %** | 1 |
|  | **45-50 %** | 1 |
