# Supplementary material for: Molecular prediction of adjuvant cisplatin efficacy in Non-Small Cell Lung Cancer (NSCLC)—validation in two independent cohorts
Source: PLoS One. 2018 Mar 22;13(3):e0194609. doi: 10.1371/journal.pone.0194609 (PMC5864030; doi:10.1371/journal.pone.0194609)
Supplement: S2 Table — Part 1 represent a univariate model per cohort, part 3 represent a multivariate model per cohort. Parts 2 and 4 represent the time-dependent analysis conducted on each cohort in a uni- and multivariate model respectively. All hazard ratios for DRP are based on a continuous score with a 50-point difference. Cohort 1 refers to the JBR.10 cohort treated with cisplatin and vinorelbine and cohort 2 refers to the RH-cohort. Adenosquamous cell carcinoma is included in the group Other in the analysis. Abbreviations: AC = adenocarcinoma; ACV = adjuvant cisplatin and vinorelbine; DRP = drug response predictor (profile), the combined cisplatin and vinorelbine predictor; Other = pleomorphic, spindle cell, high grade mucoepidermoid carcinoma and adenosquamous cell carcinoma; SCC = squamous carcinoma. (DOCX) [file pone.0194609.s005.docx]

**S2 Table. U133 CIS predictor, endpoint DSS and OS.**

|  | | | | **Endpoint** | | | | | |
| --- | --- | --- | --- | --- | --- | --- | --- | --- | --- |
|  |  |  |  | **Disease-Specific Survival** | | | **Overall Survival** | | |
|  |  |  |  | **Hazard Ratio** | **95% Confidence Limit for Hazard Ratio** | **P-value** | **Hazard Ratio** | **95% Confidence Limit for Hazard Ratio** | **P-value** |
| **Model** | **Cohort** | **Parameter** | **Level** | 0.37 | (0.12-1.15) | 0.09 | 0.47 | (0.17-1.27) | 0.14 |
| **1: Univariate** | **1** | **DRP 50-point difference** |  |  |  |  |  |  |  |
|  | **2** | **DRP 50- point difference** |  | 0.59 | (0.22-1.60) | 0.30 | 0.82 | (0.34-1.99) | 0.66 |
| **2: Univariate Time Dependent** | **1** | **DRP < 3 years** |  | 0.41 | (0.10-1.59) | 0.20 | 0.43 | (0.12-1.56) | 0.20 |
|  |  | **DRP > 3 years** |  | 0.32 | (0.04-2.24) | 0.25 | 0.53 | (0.11-2.58) | 0.43 |
|  | **2** | **DRP < 3 years** |  | 0.14 | (0.03-0.59) | **0.0076** | 0.15 | (0.04-0.59) | **0.0071** |
|  |  | **DRP > 3 years** |  | 2.31 | (0.60-8.86) | 0.22 | 2.80 | (0.91-8.61) | 0.07 |
| **3: Multivariate model** | **1** | **Gender** | **Female** | 0.85 | (0.31-2.32) | 0.75 | 0.73 | (0.31-1.76) | 0.49 |
|  |  | **Age 10-year difference** |  | 1.59 | (0.94-2.71) | 0.09 | 1.59 | (0.99-2.56) | 0.055 |
|  |  | **DRP 50-point difference** |  | 0.52 | (0.18-1.55) | 0.24 | 0.62 | (0.24-1.59) | 0.32 |
|  |  | **Histology (vs. AC)** | **Other** | 1.34 | (0.35-5.18) | 0.67 | 0.84 | (0.23-3.05) | 0.79 |
|  |  |  | **SCC** | 0.38 | (0.13-1.08) | 0.07 | 0.30 | (0.11-0.77) | **0.013** |
|  |  | **Stage (vs. 1)** | **2** | 1.79 | (0.78-4.15) | 0.17 | 1.22 | (0.58-2.57) | 0.59 |
|  | **2** | **Gender** | **Female** | 0.81 | (0.40-1.64) | 0.55 | 0.90 | (0.47-1.74) | 0.76 |
|  |  | **Age 10-year difference** |  | 1.55 | (0.98-2.43) | 0.06 | 1.57 | (1.02-2.40) | **0.039** |
|  |  | **DRP 50-point difference** |  | 0.54 | (0.17-1.71) | 0.30 | 0.77 | (0.27-2.19) | 0.62 |
|  |  | **Histology (vs. AC)** | **Other** | 0.46 | (0.17-1.27) | 0.13 | 0.48 | (0.19-1.23) | 0.13 |
|  |  |  | **SCC** | 0.19 | (0.04-0.85) | **0.030** | 0.34 | (0.11-1.04) | 0.058 |
|  |  | **Stage (vs. 1)** | **2** | 2.41 | (1.03-5.67) | **0.044** | 2.91 | (1.31-6.45) | **0.0084** |
|  |  |  | **3** | 2.69 | (1.10-6.53) | **0.029** | 3.51 | (1.50-8.19) | **0.0037** |
| **4: Multivariate Time-dependent analysis per study** | **1** | **Gender** | **Female** | 0.85 | (0.31-2.31) | 0.75 | 0.73 | (0.31-1.76) | 0.49 |
|  |  | **Age 10-year difference** |  | 1.60 | (0.94-2.72) | 0.09 | 1.59 | (0.99-2.56) | 0.055 |
|  |  | **Histology (vs. AC)** | **Other** | 1.34 | (0.35-5.19) | 0.67 | 0.84 | (0.23-3.05) | 0.79 |
|  |  |  | **SCC** | 0.37 | (0.13-1.08) | 0.07 | 0.30 | (0.11-0.77) | **0.013** |
|  |  | **Stage (vs. 1)** | **2** | 1.79 | (0.77-4.15) | 0.17 | 1.22 | (0.58-2.57) | 0.59 |
|  |  | **DRP < 3 years** |  | 0.58 | (0.16-2.11) | 0.41 | 0.61 | (0.18-2.01) | 0.41 |
|  |  | **DRP > 3 years** |  | 0.42 | (0.062-2.85) | 0.37 | 0.63 | (0.14-2.79) | 0.54 |
|  | **2** | **Gender** | **Female** | 0.85 | (0.42-1.74) | 0.65 | 0.94 | (0.48-1.83) | 0.86 |
|  |  | **Age 10-year difference** |  | 1.60 | (1.01-2.54) | **0.046** | 1.61 | (1.04-2.49) | **0.032** |
|  |  | **Histology (vs. AC)** | **Other** | 0.47 | (0.17-1.29) | 0.14 | 0.49 | (0.19-1.24) | 0.13 |
|  |  |  | **SCC** | 0.20 | (0.04-0.87) | **0.032** | 0.35 | (0.12-1.07) | 0.06 |
|  |  | **Stage (vs. 1)** | **2** | 2.43 | (1.05-5.59) | **0.037** | 2.92 | (1.34-6.33) | **0.0067** |
|  |  |  | **3** | 2.83 | (1.17-6.85) | **0.021** | 3.70 | (1.59-8.57) | **0.0023** |
|  |  | **DRP < 3 years** |  | 0.10 | (0.02-0.51) | **0.0061** | 0.09 | (0.02-0.47) | **0.0044** |
|  |  | **DRP > 3 years** |  | 3.17 | (0.66-15.30) | 0.15 | 4.04 | (1.05-15.63) | **0.043** |
