# Supplementary material for: Molecular prediction of adjuvant cisplatin efficacy in Non-Small Cell Lung Cancer (NSCLC)—validation in two independent cohorts
Source: PLoS One. 2018 Mar 22;13(3):e0194609. doi: 10.1371/journal.pone.0194609 (PMC5864030; doi:10.1371/journal.pone.0194609)
Supplement: S3 Table — Multivariate analysis of the miRNA cisplatin predictor in a time-dependent model with a cut-off of 3 years. The predictor is scored as a continuous variable and the hazard ratio estimates are for a 50-point difference. Adenosquamous cell carcinoma is included in the group Other in the analysis. Abbreviations: AC = adenocarcinoma; Other = pleomorphic, spindle cell, high grade mucoepidermoid carcinoma and adenosquamous cell carcinomas; SCC = squamous carcinoma. (DOCX) [file pone.0194609.s006.docx]

**S3 Table. Mir CIS predictor, endpoint OS.**

| **Parameter** |  | **Hazard Ratio** | **95% Confidence Limit for Hazard Ratio** | **P-value** |
| --- | --- | --- | --- | --- |
| Prediction < 3 years |  | 1.13 | (0.40-3.28) | 0.82 |
| Prediction > 3 years |  | 1.86 | (0.65-5.24) | 0.25 |
| Stage vs.1 | 2 | 2.82 | (1.26-6.27) | **0.011** |
| Stage vs. 1 | 3 | 3.97 | (1.67-9.41) | **0.0017** |
| Age 10-year difference |  | 1.59 | (1.02-2.46) | **0.039** |
| Gender |  | 1.13 | (0.58-2.18) | 0.72 |
| Histology vs AC | OTHER | 1.43 | (0.47-4.33) | 0.53 |
|  | SCC | 0.38 | (0.12-1.21) | 0.10 |
